# Book Notices

**Published:** 1856-02

**Authors:** 


### BOOK NOTICES.

*Letters to a Young Physician just entering upon Practice.* By James Jackson, M. D., LL. D., Professor Emeritus of the Theory and Practice of Physic in the University at Cambridge; Corresponding Member of the Academy of Medicine at Paris, &c., &c. Boston: Phillips, Sampson and Company. New York: J. C. Derby, 1855.

In the dedication to John C. Warren, the author explains the objects of the work, and the materials out of which it has been constructed. It was not designed to be a systematic work, "but," says the writer, "I thought it possible, availing myself of the form and license of letters, to give whatever useful thing experience could furnish." The book will be read with interest by the junior members of the profession, as containing the views of an old and eminent practitioner, untrammled by authors or the doctrines of the day.

There is but little discussion about theories, but a storehouse of valuable precepts and practical doctrines for the management of diseases. We make a few extracts which serve to show the style of the author. On page 67, in reference to the treatment of epilepsy, we find the following:—

"I believe that, in a certain proportion of the cases of epilepsy, the disease is susceptible of relief, so as not to return without the operation of some powerful cause. This relief is not to be attained by any medicine with which I am acquainted, but by diet. The diet, which I have directed with success, has been almost purely vegetable. I have directed an entire abstinence from flesh and fish, but have allowed the use of milk and butter, and occasionally of eggs. I have thought it necessary to use these last watchfully; that is, in moderate quantity, mixed with farinaceous substances, as in puddings, and at those times only when the health was at the best. I was led into the use of this diet gradually; I hardly remember by what steps. Under the use of it I have seen many recoveries; yet, in the larger proportion of cases, it has failed. But to this I must add, that I have not known an instance where the patient has ultimately recovered after trying this diet without success. Now, of the cases of which it has failed, probably every one employed various remedies, not only under my directions, but under that of others, and those, skilful physicians. Every medical man knows that in this dreadful disease, when long continued, the patient or his friends resort to every one who holds out a prospect of relief, whether he be a regular or an irregular practitioner.

"It is proper to add that, when directing this diet, I have also directed that every possible precaution should be taken to guard against fright, or agitation of mind, of any kind; against over-fatigue, excessive indulgence in food, and all other exciting causes. I will give one instance, in reference to this point, among several which I have known. A gentleman brought me his son, a boy about twelve years of age, whose health was good in other points, but in whom epilepsy occurred under its most certain character. This was in February. The gentleman lived in the country, and I did not see him till the next September. He then told me that his son had only one attack; and that in August, after an excessive indulgence in green apples. The apples were subsequently thrown off. This was nearly twenty years ago, and the patient has never had another attack."

On pages 167 and 168 we find the follwing, in reference to rheumatism:—

"*Rheumatism* is a very large box, and contains quite a variety of things; and these things do not all agree, either as to their material, or their form. This is conceded at once as to the acute and chronic. The acute disease, known as *rheumatic fever*, has a pretty distinct character; its form and pressure are such that a man, who has once endured them, does not easily forget them. If any good is to be done in this disease, it must be at the very beginning; just as I have endeavored to show about all diseases, especially such as are acute. The chance in this disease is not so good as in some others. Free evacuations and low diet from the first are, I think, certainly useful, though they do not jugulate the disease. But there is a natural relief in two or three weeks, and then, patient and doctor are too apt to think the disease is going away. Not so; if ever, very rarely. At this stage medicine will not do much, if anything; but care may do much. Not the care which keeps a man smothered up, such as to increase the excessive perspiration which belongs to the disease. Let the patient be as lightly covered as is consistent with his present comfort; that will be enough. But the care must be in avoiding any great effort of body or mind, in maintaining calmness and tranquility, and in moderate diet. Give enough milk and bread, or some other vegetable article, to support the patient; but withhold animal food. If the disease is not checked at the beginning, avoid all strong medicines, except opium for severe pain. Keep the bowels open, and be watchful as to diet. If anything has been useful in my hands *after the first few days of the disease*, it is the sulphate of quinia. This article, taken as freely as it is for intermittent fever, appears sometimes to arrest the disease. It is not, however, as certain as was believed by Dr. Haygarth in respect to the cinchona itself."

In the last letter, on the treatment of typhoid fever, Dr. Jackson gives the following statistics as to the effects of emetics, from which it would seem that this method of treatment if resorted to early in the disease, may diminish the mortality and hasten convalescence.

"Without going into all particulars, I will take what relates to the effects of emetics. This report was grounded on the cases of that disease in the hospital from 1822 to 1835, inclusive. From this it appears (pp. 7 and 8) that on a fair estimate there was one death in about eight cases.

| | |
|---|---|
| Among those admitted to the hospital in the first two weeks of the disease, one hundred and fifty took emetics before or after admission; of these one hundred and fifty, thirteen died, being | 1 in 11.53 |
| In the same period eighty were admitted who did not take emetics; of these ten died, being | 1 in 8.00 |

The difference is very striking. But of the one hundred and fifty who took emetics, some took them earlier and some later in the disease. It has been thought that the earlier this and other active and depletory remedies are administered, the greater the benefit. See how far this is confirmed by the same report.

| | |
|---|---|
| Fifty-nine entered the first week of the disease, and took emetics in that week; four of these died, being | 1 in 14.75 |
| Thirty-one entered the same week, and did not take emetics; of these three died, being | 1 in 10.33 |
| Ninety-one entered the second week, and took emetics either before or after admission; of these nine died, being | 1 in 10.11 |
| Forty-eight entered the same week, and did not take emetics; of these seven died, being | 1 in 6.85 |

The advantage was on the side of those, who took emetics; but more decided as to those who entered the first week, than as to those who entered the second. The last had not probably been so well nursed in the first week as the others, But also they had not on an average taken the emetic so early; and that, no doubt, made a difference in favor of those entering the first week. My own experience taught me long ago that emetics were most useful then within the first three days of the disease. This is confirmed by the hospital cases. It appears from the report that thirty-two patients took emetics within the first three days of the disease; of these one died, 1 in 32

Twenty-seven took emetics within the last four days of the first week; of these three died, being 1 in 6

Undoubtedly these last numbers, relative to those who took emetics in the last four days of the first week, are less favorable than would be found if the number was larger; for the proportion of deaths is greater than in those who entered the second week, and took emetics, and of whom the larger part, no doubt, took their emetics at a later period of the disease than the above twenty-seven.

If, now, we inquire what was the effect on the duration of the disease in those, who took emetics and recovered, it does not seem to have been much if anything to the advantage of those taking emetics later than the third day. But as to those who took emetics on either of the first three days the benefit is unequivocal.

Of those who took emetics on the first day of the disease, the average day of convalescence was the 14.66

Of those who took emetics on the second day, the average day of convalescence was the 15.32

Of those who took emetics on the third day, the average day of convalescence was the 16.46

While of those who took emetics on the fourth, fifth, and sixth days of the disease, and recovered, the average day of convalescence was the 19.45

It cannot surely be attributed to accident that these results were so favorable to those, who took emetics, among cases not selected but taken as they come, through many successive years."

J.

---

*A Treatise on Epidemic Cholera.* By HORATIO GATES JAMESON, Senr., M. D., Member of the Medical and Surgical Faculty of Maryland, &c., &c. Philadelphia: Lindsay & Blakiston, 1855.

The author believes that cholera is the product of a new combination of electricity—that the first impression is made upon the mucous membrane of the alimentary canal, a surface usually possessed of but little sensibility, but liable to diseased

ıction, in which it becomes in the highest degree sensative and painful. The disturbance of the innervation of the parts gives rise to diarrhœas and dysentry in their epidemic form, or to true cholera, according to the impressibility of the membrane and the amount of irritation to which it is subjected. In these morbid conditions of the splanchnic nervous system, according to the author, "an innocent article taken into the stomach may give rise to cholera, which shall assume its highest intensity, as respects sufferings, in a few minutes." According to our experience, the first stages of cholera are not generally attended with pain, and by this fact the patients are lulled into a fancied security until a serious change in the fluids, and perhaps also of the solids of the body is effected, the evidence of which is felt in the severe muscular convulsions and depression of the organic life forces. The following is a summary of the treatment recommended in the body of the work :—

"The prelude to cholera is a fault of function in the abdominal viscera, as manifested in the epidemic diarrhœas that preceded cholera lethalis. What is the most rational indication? We say the most rational indication is to arrest the incubating tendencies, which is to be done by correcting the vicious secretions. But how is this to done?

"1st. The terminal nerves on the surface of the stomach are in a state of increased and morbid sensitiveness. Opium would seem to be the antidote here, but we find in the early stages, or mild cases, carminatives, as soda and sassafras, camphor, chloroform, cold water, ice, &c., will arrest the diarrhœa; and, this may be ascribed to these articles bringing the nervous influence, so to speak, to its senses; and doing its more normal part in the atomic, plastic, and eliminative functions, which are now brought into healthful play; and we have restored the secretion.

"2d. Whenever the nervous structures are highly sensitive, and there is agonizing pains about the præcordia, or violent or general spasms, we shall find the fault of function more deeply laid; and here we must combine our soothing agent with one that will influence the fault of assimilation in the atomics; for this purpose calomel and opium are the most reliable agents.

"3d. Is there profuse discharges upwards and downwards, which will speedily prostrate the general system,—four or five grains of opium, given according to the urgency of the case, at one, two, or three doses; never at longer intervals than one or two hours, if given at the commencement of the lethargic stage (as seen in listlessness, weak voice, indifference, &c.,) will arrest the diarrhœa, and we have now time to attend to the urinary secretion; and for this purpose eight or ten grain doses of nitrate pot. combined with five or ten grain doses of calomel, should there seem to be deep-laid atomic aberration.

"4th. Cases occur where, after eating, and sometimes from over exertions, &c., persons are brought into one blazing agony of suffering; here the lancet is the remedy; and, for its successful application, bleeding must be carried on till there is complete relief from pain, and a more florid appearance of the blood.

"5th. While internal remedies are employed, the application of hogslard will be found an important remedy; a cup of hot water can be carried to the bedside, with a small one in the water and the lard in a small cup, will be found a convenient way of applying it: the patient being wiped dry, is to be freely rubbed with the lard. We have elsewhere treated fully of the treatment of cholera, but we have thought proper to say thus much, by which we hope to show the reader that according to the different phases, stages, and circumstances, remedies are to be individually applied. But we have deemed it a special duty to advise, that, according to our observation and experience, seasons modify epidemic diseases; and, by especial attention to this truth, at the beginning of an epidemic, we may soon establish a preferable treatment, and then manage our case with comparative satisfaction to ourselves and to the greater safety of our patients.

"6th. The vast number of remedies that have obtained celebrity for the cure of cholera, and the readiness with which it is seen to yield to remedies, in its early stages; and, now and then in cases more profound, goes to establish the belief, that the predisposing cause is but a feeble poison; and, experience abundantly proves, that precautions, and mild prophylactics are much to be relied on.

"Dr. Rush once said to us, the reverse of *quod cito fit cito perit*, is true in medicine; but in the treatment of cholera lethalis, what we do, we must do quickly, or decomposition of the blood will lead to the grave."

J.

---

*The Diseases of the Heart and the Aorta.* By WM. STOKES, Regius Professor of Physic in the University of Dublin; author of the Treatment and Diagnosis of Diseases of the Chest, &c. Philadelphia: Lindsay & Blakiston, 1855.

We are under obligations to the publishers for the valuable work, the title of which is given above. It contains the opinions of the author, derived from an experience of more than a quarter of a century in reference to the rational application of cardiac pathology and physical diagnosis to practical medicine. To an abundance of material for prosecuting this branch of medical study, the author has brought an untiring industry, and a zeal for the developement of truth, honorable alike to the man and the profession.

The volume contains 710 pages, and will be found a valuable aid to the student and practitioner in the investigation and management of this class of diseases.

For sale by D. B. Cooke & Co., Chicago, Ill. J.

